# Investigation of carotid intima-media thickness in patients with schizophrenia

**DOI:** 10.1186/s12888-024-05496-7

**Published:** 2024-01-17

**Authors:** Yaşar Kapıcı, Olga Bayar Kapıcı, Sabri Abuş, Mehmet Hamdi Örüm, Selçuk Ayhan, Mehmet Bozkurt, Bilal Özer, Atilla Tekin

**Affiliations:** 1 Psychiatry Department, Adıyaman University Faculty of Medicine, Adıyaman, Turkey; 2 Radiology Department, Adıyaman Training and Research Hospital, Adıyaman, Turkey; 3 Cardiology Department, Adıyaman University Faculty of Medicine, Adıyaman, Turkey; 4 Psychiatry Department, Elazığ Mental Health and Diseases Hospital, Elazığ, Turkey; 5 Cardiology Department, Adıyaman Training and Research Hospital, Adıyaman, Turkey; 6 Cardiology Department, Kahta State Hospital, Adıyaman, Turkey; 7 Radiology Department, Kahta State Hospital, Adıyaman, Turkey; 8 Psychiatry Department, Adıyaman University Faculty of Medicine, Adıyaman, Turkey

**Keywords:** Schizophrenia, Carotid intima-media thickness, Atherosclerosis, Electrocardiogram

## Abstract

**Background:**

Patients with schizophrenia (SCZ) have a higher risk of cardiovascular diseases than the average population. Early diagnosis of SCZ patients with subclinical atherosclerosis is great importance in reducing cardiovascular morbidity and mortality. The aim of this study was to investigate some clinical risk factors for atherosclerosis in patients with SCZ.

**Methods:**

Fifty-one SCZ patients (20 females, 31 males) and 55 healthy controls (HCs) (25 females, 30 males) were included in the study. Electrocardiography (ECG), lipid parameters, hemogram, and biochemistry values of the participants were taken. Low-density lipoprotein (LDL), high-density lipoprotein (HDL), fasting triglycerides, and total cholesterol were measured. The arrhythmogenic index of plasma (AIP) was analyzed. The recorded right and left carotid intima-media thickness (CIMT) measurements by carotid ultrasonography were scanned.

**Results:**

QT interval (*p* = 0.035), CIMT-left (*p* = 0.008), CIMT-right (*p* = 0.002), fasting triglyceride (*p* = 0.005), AIP (*p* = 0.005) in the SCZ group compared to HCs (< 0.001) was statistically higher, while HDL (*p* = 0.003) was statistically lower. Smoking rates, QT interval (*p* = 0.035), CIMT-left (*p* = 0.008), and CIMT-right (*p* = 0.002) were significantly higher in the the SCZ group than in the HCs. According to odds ratios, individuals with SCZ have a 6.3-fold higher smoking rate. According to Pearson correlation analysis, CIMT-left was positively correlated with age and QT interval (*r* = 0.568, *p* < 0.001 and *r* = 0.589, *p* < 0.001, respectively). CIMT-right value was also positively correlated with age and QT interval (*r* = 0.533, *p* < 0.001 and *r* = 0.555, *p* < 0.001, respectively). QT interval positively and significantly predicted CIMT-left and CIMT-right (*p* < 0.001, β = 0.549 and *p* = 0.001 and β = 0.506 accordingly).

**Conclusion:**

In this study, a close relationship was found between the QT interval and CIMT in SCZ patients. This finding could be valuable for using an easy-to-calculate data such as QT in place of a laborious test such as CIMT.

## Introduction

Schizophrenia (SCZ) is a chronic disabling mental illness with a 2–3 times higher chance of death than the overall populace. The lifetime prevalence of SCZ is approximately 1%. Furthermore, to the chronicity of the condition, the patients diagnosed with SCZ have a greater likelihood than the normal population for a variety of physical ailments, including diabetes, obesity, and cardiometabolic disease. Because of these accompanying chronic diseases, the life quality and life expectancy decrease in the patients diagnosed with SCZ [1].

Cardiovascular diseases, which are the main reasons of disability and premature death globally, are more prevalent in the patients diagnosed with SCZ. Nevertheless, they are often left undiagnosed. Researchers have demonstrated that factors associated with cardiovascular conditions such as overweight, diabetes, metabolic syndrome, elevated blood pressure, hyperlipidemia, tobacco use, and a lack of physical activity are more frequent in the patients diagnosed with SCZ than in the average community. Treating accompanying physical conditions in the patients diagnosed with SCZ improves the overall wellbeing and reduces premature mortality. Therefore, an important aspect of the care of the patients diagnosed with SCZ is the provision of physical medical care alongside psychiatric treatment [2].

Families and caregivers should follow up on additional medical conditions that may accompany the psychiatric symptoms of the patients diagnosed with SCZ. Family health centers and community mental health centers are health institutions that mediate this close follow-up. Routine examinations and measurements of patients are carried out here. The subsequent parameters have been suggested to help in rapid detection of accompanying medical conditions in the surveillance of the patients diagnosed with SCZ: blood glucose levels, body mass index, fasting triglyceride levels, high-density lipoprotein cholesterol, low-density lipoprotein cholesterol and blood pressure. Possible changes in the measurements listed above are consulted with the relevant internal branches, and recommendations are followed in these centers [3].

Carotid intima-media thickness (CIMT) is a well-studied indicator of developing atherosclerosis. Over the past few years, CIMT has gained attention as a potential determinant of cardiac events. Its reliability as an appropriate assessment of atherosclerotic disease has been confirmed in numerous investigations in a variety of populations [4]. CIMT has been repeatedly investigated in non-psychiatric diseases. Although CIMT has not been sufficiently investigated in psychiatric diseases, various studies have been conducted. Santos et al. measured CIMT in patients with anxiety and depressive symptoms and found that CIMT increased as disease severity increased. The authors found a positive correlation between increased subclinical atherosclerosis and anxiety/depression symptoms [5]. Pizzi et al. found an independent relationship between depression symptoms and CIMT [6]. Studies investigating CIMT in SCZ are insufficient.

The patients diagnosed with SCZ have a greater likelihood of heart conditions and acute heart attacks than the average person; hence, they have a high chance of death from these ailments. In accordance with the World Health Organization (WHO), eight preventable causes (excessive alcohol consumption, smoking, hypertension, obesity, hypercholesterolemia, hyperglycemia, inadequate fruit and vegetable consumption, and sedentary lifestyle) are blamed for 61% of annual cardiovascular mortality. The WHO also noted that minimal contact to factors related to mortality could extend global lifespan by nearly five years. It is widely accepted that these comorbidity and mortality factors are more prevalent in the patients diagnosed with SCZ, and treating at an early stage in the patients diagnosed with SCZ with subclinical atherosclerosis reduces comorbidity and mortality [7].

All in all, it can be said that the patients diagnosed with SCZ have a higher risk for cardiovascular and metabolic abnormalities than the general population. Although many risk factors for cardiovascular and metabolic problems have been reported in patients diagnosed with SCZ, CIMT has not been investigated as an atherosclerosis indicator. It has been anticipated that CIMT in patients diagnosed with SCZ may differ from healthy individuals. Therefore, the aim of the study is to compare CIMT between the patients diagnosed with SCZ and healthy controls. To this end, the presence of subclinical atherosclerosis in the patients diagnosed with SCZ will be investigated by retrospectively comparing CIMT, hemogram, biochemistry, lipid panel, and electrocardiogram (ECG) results of the patients diagnosed with SCZ with the control group without any cardiac or psychiatric disease.

## Material and method

Permission for this study was obtained from the Non-Invasive Clinical Research Ethics Committee of Fırat University Faculty of Medicine (Approval Date: 15/12/2022, IRB Number: 2022/ 15 − 08). The patients diagnosed with SCZ may apply to the cardiology outpatient clinic for palpitations, shortness of breath, or orthostatic hypotension. Routine hemograms, biochemistry, and lipid results of these patients are examined. ECG is taken for all patients who apply to the cardiology outpatient clinic, and these results are archived. In addition, carotid Doppler ultrasonography can be performed for patients with a high risk of atherosclerosis, such as SCZ. In this study, the data of the patients diagnosed with SCZ will be compared with those who applied to the cardiology outpatient clinic but did not receive any diagnosis and had carotid Doppler results. The study group will be composed of cases who applied to Kahta State Hospital’s cardiology outpatient clinic. Patients with hypertension, diabetes mellitus, known coronary artery disease, hypothyroidism, hyperthyroidism, and valve disease were not included in the patient group. Care was taken to ensure that the control group had no known organic or psychiatric disease.

The sample size of the study was calculated using G*Power 3.1.3 (Duseldorf, Germany). Accordingly, the minimum sample for null hypothesis was found 44. (Cohen’s d = 0.2, alpha = 0.05, power = 0.8). Fifty-one patients diagnosed with SCZ (20 females, 31 males) and 55 healthy controls (HCs) (25 females, 30 males) were included in the study. Electrocardiography (ECG), lipid parameters, hemogram, and biochemistry values of the participants were taken. Right and left CIMT measurements were made by carotid ultrasonography. Heart rate, QT interval, corrected QT interval, and QRS width were evaluated in ECG. In the lipid panel, low-density lipoprotein (LDL), high-density lipoprotein (HDL), fasting triglycerides, and total cholesterol were measured. The arrhythmogenic index of plasma (AIP) was analyzed. AIP was calculated by taking the logarithm of the ratio of triglyceride to HDL. The body mass index (BMI) of the participants was measured.

### Statistical analysis

SPSS version 22 package program was used for statistical analysis. The values obtained in the study were given as mean ± standard deviation. The Student’s t-test was used for comparisons between groups if the measured data showed normal distribution. Otherwise, the Mann-Whitney U test was used. The chi-square test was used to compare categorical data. Pearson correlation analysis, linear regression analysis, and binary logistic regression analysis were applied. A value of *p* < 0.05 was considered statistically significant.

## Results

The comparison of sociodemographic data, ECG records and CIMT measurements of the the patients diagnosed with SCZ and the HCs are shown in Table-1. The mean age was 36.64 ± 7.81 years in the SCZ group and 38.50 ± 7.29 years in the HCs group. There was no significant difference between the groups regarding gender distribution and mean age (*p* = 0.117 and *p* = 0.418, respectively). Smoking rates, QT interval (*p* = 0.035), CIMT-left (*p* = 0.008), and CIMT-right (*p* = 0.002) were significantly higher in the the SCZ group than in the HCs. According to odds ratios, individuals with SCZ have a 6.3-fold higher smoking rate.

**Table 1:** Comparison of Sociodemographic Features and Electrocardiographic Parameters of Patients Diagnosed with Schizophrenia and Healthy Controls

	SCZ (*n* = 51) M ± SD or n (%) or Med. (Min:Max)	HCs (*n* = 55) M ± SD or n (%) or Med. (Min:Max)	Statistic	p	OR	%95 CI Lower/Upper
**Age**	37.24 ± 7.22	38.26 ± 7.46		0.685^1^		0.30 / 2.9
**Gender**				0.478^2^	1.224	0.23 / 2.71
**Female**	20 (39.2)	25 (45.6)				
**Male**	31 (60.8)	30 (54.4)				
**Smoking**	38 (74.5)	28 (50.9)		***0.035*** ^***2***^	***6.302***	***5.306 / 9.2***
**BMI**	26.9 ± 4.9	26.2 ± 4.4		0.684^1^		0.023 / 19.4
**Systolic blood pressure, mmHg**	123.4 ± 7.26	117.6 ± 6.46		0.522^1^		0.135 / 46.8
**Diastolic blood pressure, mmHg**	73.4 ± 5.18	69.6 ± 4.64		0.604^2^		0.261 / 8.1
**LVEF, %**	62.5 ± 4.6	64.6 ± 3.8		0.816^3^		0.024 / 4.6
**Heart rate, bpm**	82.4 ± 10.36	79.6 ± 11.86		0.206^1^		0.015 / 9.7
**QRS, msec**	91.27 ± 9.61	92.91 ± 9.63	0.564	0.576^1^		0.109 / 4.1
**QT, msec**	367.82 ± 34.40	346.64 ± 29.91	-2.179	0.035^1^		0.001 / 4.9
**QTc, msec**	407 (377–472)	410 (376–444)	0.096	0.925^3^		0.406 / 3.7
**CIMT-left**	0.86 ± 0.15	0.76 ± 0.06	-2.873	***0.006*** ^***1***^		***7.190 / 11.181***
**CIMT-right**	0.87 ± 0.15	0.75 ± 0.06	-3.325	***0.002*** ^***1***^		***12.760 / 31.318***

SCZ, schizophrenia; HCs, healthy controls; LVEF, left ventricular ejection fraction; QTc, corrected QT interval; CIMT, carotid intima media thickness

^1^Independent t test was used. ^2^Chi-square test was used. ^3^Mann-Whitney U test was used. *p* < 0.05 was accepted as statistically significant. Multivariable logistic regression model was created (Cox & Snell R^2^: 0.46; Nagelkerke R^2^: 0.62)

Binary logistic regression analysis was applied to reveal the relationship between CIMT-right and the groups (SCZ & HCs). According to the binary logistic regression analysis, the sensitivity of CIMT-right related to the determining the participants who was involved in SCZ & HCs groups was 68.2, and the specificity was 68.2% (Beginning block, -2 log-likelihood = 60.997^a^, *p* = 0.002; Block one, -2 log-likelihood = 49.490^a^; Cox & Snell R^2^ = 0.230; Nagelkerke R^2^ 0.307; Hosmer and Lemeshow Test *p* = 0.553; Constant *p* = 0.006, CIMT-right *p* = 0.007, Constant B=-8.865, CIMT-right B = 11.09).

Comparison of laboratory parameters of the patients diagnosed with SCZ and HCs are shown in Table-2. QT interval (*p* = 0.035), basophil count (*p* = 0.011), fasting triglyceride (*p* = 0.005), AIP (*p* = 0.005) in the SCZ group were significantly higher, while HDL (*p* = 0.003) was significantly lower compared to HCs.

**Table 2:** Comparison of Laboratory Parameters of Patients Diagnosed with Schizophrenia and Healthy Controls

	SCZ (*n* = 51) M ± SD or n (%) or Med. (Min:Max)	HCs (*n* = 55) M ± SD or n (%) or Med. (Min:Max)	Statistic	p
**Hemoglobin, mg/dl**	14.23 ± 2.26	14.19 ± 1.82	-0.059	0.953^1^
**Urea, mg/dl**	27.91 ± 8.33	28.87 ± 8.42	0.378	0.707^1^
**Creatinine, mg/dL**	0.80 ± 0.12	0.76 ± 0.12	-0.958	0.344^1^
**Albumin, mg/dL**	4.37 ± 0.26	4.49 ± 0.27		0.856^1^
**Neutrophil, 10** ^**6**^ **/µL**	4.87 ± 1.76	4.81 ± 1.41	-0.114	0.909^1^
**Lymphocyte, 10** ^**3**^ **/µL**	2.39 ± 0.91	2.43 ± 1.06	0.129	0.898^1^
**Platelet, 10** ^**3**^ **/µL**	268.46 ± 76.39	254.56 ± 62.46		0.231^1^
**Monocyte, 10** ^**3**^ **/µL**	0.56 ± 0.30	0.54 ± 0.62	-0.170	0.866^1^
**Eosinophil, 10** ^**3**^ **/µL**	0.07 (0-0.34)	0.06 (0-0.41)	-0.681	0.496^2^
**Basophil, 10** ^**3**^ **/µL**	0.08 (0-0.43)	0.04 (0-0.14)	-2.431	***0.015*** ^***2***^
**Fasting Triglycerid, mg/dL**	188.36 ± 95.77	114.59 ± 67.87	-2.948	***0.005*** ^***1***^
**Total Cholesterol, mg/dL**	185.36 ± 37.43	166.59 ± 37.93	-1.652	0.106^1^
**LDL-C, mg/dL**	92.86 ± 28.09	81.40 ± 29.41	-1.321	0.194^1^
**HDL-C, mg/dL**	51.72 ± 12.20	62.27 ± 9.64	*3.179*	***0.003*** ^***1***^
**CRP, mg/dl**	0.2 (0.2–9.5)	0.2 (0.2-1)	-0.952	0.341^2^
**NLR**	2.11 (0.88–8.33)	2.13 (0.88–4.28)	-0.376	0.707^2^
**PLR**	110.5 (62.52-438.33)	96.4 (46.3-378.76)		0.086^2^
**MLR**	0.23 (0-0.79)	0.22 (0-1.45)	-0.704	0.481^2^
**MHR**	0.01 (0-0.03)	0.008 (0-0.05)	-2.253	***0.024*** ^***2***^
**CAR**	0.04 (0.04–2.16)	0.05 (0.04–0.26)	-1.494	0.135^2^
**AIP**	0.52 ± 0.26	0.21 ± 0.24	-4.059	***< 0.001*** ^***1***^

SCZ, schizophrenia; HC, healthy controls; WBC, white blood cell; LDL-C, low-density cholesterol; HDL-C, high-density cholesterol; CRP, c-reactive protein; NLR, neutrophil lymphocyte ratio; PLR, platelet lymphocyte ratio; MLR, monocyte lymphocyte ratio; MHR, monocyte HDL-C ratio; CAR, CRP albumin ratio; AIP, atherogenic index of plasma

^1^Independent t test was used. ^2^Mann-Whitney U test was used. *p* < 0.05 was accepted as statistically significant

Binary logistic regression analysis was applied to reveal the relationship between the parameters found to be significant in Table 2 and the groups (SCZ & HCs). Before creating the model, significant variables were examined one by one using binary logistic regression analysis. MHR, which was found to be significant in Table 2, did not yield significant results with binary logistic regression analysis and was therefore removed from the model. Basophil count, fasting triglyceride, HDL-C, and AIP were included in the model. According to the binary logistic regression analysis, the sensitivity of parameters (basophil count, fasting triglyceride, HDL-C, AIP) related to the determining the participants who was involved in SCZ & HCs groups was 63.6, and the specificity was 72.7% (Beginning block, -2 log-likelihood = 60.997^a^, overall *p* = 0.004; Block one, -2 log-likelihood = 42.401^a^; Cox & Snell R^2^ = 0.345; Nagelkerke R^2^ 0.460; Hosmer and Lemeshow Test *p* = 0.224; Constant *p* = 0.783, Constant B=-0.886, Constant Exp (B) = 0.412).

Pearson correlation analyzes of CIMT with age and ınflammatory parameters in the patients diagnosed with SCZ are shown in Table-3. According to Pearson correlation analysis, CIMT-left was positively correlated with age and QT interval (*r* = 0.568, *p* < 0.001 and *r* = 0.589, *p* < 0.001, respectively). CIMT-right value was also positively correlated with age and QT interval (*r* = 0.533, *p* < 0.001 and *r* = 0.555, *p* < 0.001, respectively).

**Table 3:** Pearson Correlation Analyzes of CIMT with Age and Inflammatory Parameters in Patients Diagnosed with Schizophrenia

	CIMT-left	CIMT-right
**Age**	*r* = 0.489	*r* = 0.470
	***p*** < 0.001	***p*** < 0.001
**QT**	*r* = 0.522	*r* = 0.545
	***p*** < 0.001	***p*** < 0.001
**MHR**	*r* = 0.206	*r* = 0.157
	*p* = 0.179	*p* = 0.310
**AIP**	*r* = 0.107	*r* = 0.060
	*p* = 0.489	*p* = 0.700

CIMT, carotid intima media thickness; MHR, monocyte HDL-C ratio; AIP, atherogenic index of plasma

*p* < 0.05 was accepted as statically significance

Linear regression analyzes of CIMT-left in the patients diagnosed with SCZ are shown in Table-4. When CIMT-left was taken as a dependent variable, QT interval positively and significantly predicted CIMT-left value in linear regression analysis (*p* < 0.001 and β = 0.549).

**Table 4:** Linear Regression Analyzes of CIMT-left in Patients Diagnosed with Schizophrenia

	B	Std. Error	Beta	t	p	95% CI	
						Lower	Upper
**Constant**	0.958	0.443		2.164	0.047	0.014	1.901
**Age** **QT**	-0.20 0.11	0.056 0.003	-0.57 0.591	-0.348 3.451	0.732 ***0.004***	-0.139 0.04	0.100 0.018
**Gender**	-0.001	0.001	-0.244	-1.367	0.192	-0.003	0.001
**MHR**	7.634	3.853	0.365	1.981	0.066	-0.579	15.848
**AIP** **BMI**	-0.204 -0.003	0.102 0.007	-0.367 -0.066	-2.005 -0.431	0.063 0.673	-0.421 -0.018	0.013 0.012

CIMT, carotid intima media thickness; MHR, monocyte HDL-C ratio; AIP, atherogenic index of plasma

Linear regression analyses was used. *p* < 0.05 was accepted as statically significance. (R^2^ = 0.69, F = 5.675, *p* = 0.003)

Linear regression analyzes of CIMT-right in the patients diagnosed with SCZ are shown in Table-5. When CIMT-right was taken as a dependent variable, the QT interval positively and significantly predicted the CIMT-right value in linear regression analysis (*p* = 0.001 and β = 0.506).

**Table 5:** Linear Regression Analyzes of CIMT-right in Patients Diagnosed with Schizophrenia

	B	Std. Error	Beta	t	p	95% CI	
						Lower	Upper
**Constant**	0.546	0.476		1.146	0.270	-0.469	1.560
**Age** **QT**	-0.001 0.013	0.001 0.003	-0.051 0.660	-0.274 3.679	0.788 ***0.002***	-0.02 0.005	0.002 0.020
**Gender**	0.001	0.060	0.002	0.013	0.990	-0.128	0.130
**MHR**	5.999	4.144	0.279	1.448	0.168	-2.833	14.831
**AIP** **BMI**	-0.240 0.001	0.110 0.007	-0.419 -0.006	-2.005 -0.035	0.063 0.973	-0.421 -0.016	0.013 0.015

CIMT, carotid intima media thickness; MHR, monocyte HDL-C ratio; AIP, atherogenic index of plasma

Linear regression analyses was used. *p* < 0.05 was accepted as statically significant. (R^2^ = 0.66, F = 4.960, *p* = 0.006)

## Discussion

According to the main result of our study, we found the CIMT-right, CIMT-left, and QT intervals to be significantly higher in the SCZ group than in the HCs group. In addition, we determined the QT interval as a predictor of atherosclerosis when CIMT-right and CIMT-left were taken as dependent variables. In this study, SCZ was found to cause a 9.7-fold increased risk for CIMT-left thickening and a 20.7-fold increased risk for CIMT-right thickening.

The patients diagnosed with SCZ have a higher risk of cardiovascular diseases and acute myocardial infarction than the average population. Therefore, the risk of death is increased in SCZ patients. Early diagnosis of the patients diagnosed with SCZ with subclinical atherosclerosis or subclinical left ventricular dysfunction is of great importance in reducing cardiovascular morbidity and mortality.

This study shows that the rate of smoking is higher in patients diagnosed with SCZ. There has been a documented association between smoking and SCZ. One hypothesis is that some individuals with SCZ may use smoking as a form of self-medication to alleviate symptoms or side effects of antipsychotic medications. Nicotine has been shown to have some effects on cognitive function, mood, and arousal, which could potentially provide relief from certain symptoms of SCZ [8].

The link between SCZ and atherosclerosis lies in the fact that individuals diagnosed with SCZ often have a higher risk of developing various physical health conditions, including cardiovascular diseases like atherosclerosis. Smoking, poor diet, lack of exercise and substance abuse, which are risk factors for atherosclerosis, are detected more frequently in patients diagnosed with SCZ. Some antipsychotic medications used to treat SCZ can lead to weight gain, metabolic disturbances, and an increased risk of diabetes and cardiovascular problems, all of which can contribute to atherosclerosis. Living with SCZ can be emotionally and psychologically challenging, and chronic stress may contribute to an increased risk of cardiovascular problems. People diagnosed with SCZ may face disparities in healthcare access and quality, which can result in less effective management of risk factors for atherosclerosis [9].

QT prolongation has been identified as a risk factor for cardiovascular mortality in healthy people, diabetes, and heart patients. Information in the literature suggests that prolonging the QT interval may be associated with atherosclerosis. CIMT is used to evaluate subclinical atherosclerosis. In 2013, Ünsal et al. found higher CIMT and diastolic dysfunction in the patients diagnosed with SCZ. In the same study, HDL levels were found to be significantly lower in the SCZ group compared to the control group, similar to our study [10]. Atypical antipsychotic drugs used in the patients diagnosed with SCZ may cause metabolic syndrome, insulin resistance and weight gain. This may be associated with an increased risk of cardiovascular disease in the patients diagnosed with SCZ.

Inflammation and endothelial dysfunction are common features of SCZ and atherosclerosis in increasing cardiovascular risk. Therefore, early evaluation and early treatment are essential. AIP is the molar ratio of circulating triglyceride and HDL-C concentrations. Also, AIP is inversely related to LDL particle size, and small intense LDL is very sensitive to oxidative damage and induces atherosclerotic lesions. Therefore, AIP has been proposed by the National Cholesterol Education Program as a risk factor for predicting cardiovascular events [11]. Yıldız et al. identified AIP as a marker in atherosclerosis [12].

Radu et al. examined the predictive value of endothelial inflammation markers in the onset of SCZ. They examined cellular adhesion molecules, soluble ICAM-1 and soluble-VCAM-1, which are biological markers that show the progression and consequences of endothelial inflammation and atherosclerosis. They found s-VCAM levels to be higher in the patients diagnosed with SCZ compared to the HCs group. They found s-ICAM − 1 level to be lower in the patients diagnosed with SCZ compared to the HCs group [13]. The coexistence of endothelial dysfunction and hyperlipidemia may explain the increase in CIMT values.

The QT interval has been associated with cardiac mortality and sudden death. It is known that QT interval prolongation may occur with the effect of antipsychotics used in SCZ. There are studies in the literature showing that the mean lifespan of the patients diagnosed with SCZ is 10 to 15 years shorter than the average population, and this is associated with cardiac morbidity and mortality. For this reason, the risk status of patients diagnosed with SCZ in terms of myocardial atherosclerosis can be observed by evaluating the QT interval, which is a cheaper and easily accessible ECG parameter, instead of CIMT, which is used in the evaluation of subclinical atherosclerosis in the patients diagnosed with SCZ [14]. QT prolongation is recorded as a common finding in the patients diagnosed with SCZ. The frequent relationship of this condition with antipsychotic drugs should not be overlooked. However, the fact that the antipsychotic drugs used in our study were not recorded is a limitation in this sense.

In 1999, Festa et al. investigated whether the QT interval indicates atherosclerosis in 912 non-diabetic patients with coronary artery disease. As a result of the study, it was shown that there is a positive relationship between QTc and carotid atherosclerosis. Accordingly, they suggested that the QT interval indicates subclinical atherosclerosis [15]. In a previous study, subclinical atherosclerosis was prospectively associated with coronary artery disease after a mean follow-up of 2.4 years. Accordingly, long QTc in the average population was associated with a higher risk of eventual coronary heart disease. Using markers of subclinical atherosclerotic disease may provide additional information to identify a high-risk individual who could benefit. Under clinical conditions, measurement of the QT interval may be easier to perform than CIMT measurement. Considering the strong correlation between coronary artery disease and carotid atherosclerosis, the QT interval may be a marker for both carotid and coronary atherosclerosis [16]. A similar result emerged in this study, which we planned based on the study conducted in diabetes patients. Further studies in different patient groups are needed to confirm the association of QT or QTc with atherosclerosis.

In prior studies, CIMT has been a prognostic marker for cardiovascular and cerebral events. In a recent study by the cardiovascular health study group, main carotid and internal CIMT measurements were associated with myocardial infarction and cerebral stroke in older adults [17, 18].

Our study has several limitations. The patient population could have been larger. Since antipsychotic drugs used by the patients diagnosed with SCZ may affect the QT interval, their effects should also be evaluated in the study. One of the limitations of our study is that calcium, magnesium, and thyroid hormone levels, which may affect the QT interval, were not taken into account. Therefore, prospective molecular and clinical studies that can better evaluate the relationship between QT interval and endothelial inflammation and atherosclerosis in the broader population should be conducted to understand inflammation in the patients diagnosed with SCZ better and contribute to new treatment approaches and reduce cardiovascular risk in these patients. Finally, due to the cross-sectional nature of the study, it is difficult to say that there is a definite relationship between the variables.

## Conclusion

In this study, a close relationship was found between the QT interval and CIMT in the patients diagnosed with SCZ. This finding could be valuable for using an easy-to-calculate data such as QT in place of a laborious test such as CIMT. In groups such as the patients diagnosed with SCZ who may have decreased functionality and treatment rejection, being able to obtain findings in the name of atherosclerosis with only ECG may be meaningful in terms of planning lifestyle changes in patients. The relationship between QT and CIMT can be examined according to the antipsychotic drug group used in future studies.

## Data Availability

The datasets used and analyzed during the current study are available from the corresponding author on reasonable request.
